# Haploinsufficiency of *BMP4* and *OTX2* in the Foetus with an abnormal facial profile detected in the first trimester of pregnancy

**DOI:** 10.1186/s13039-017-0351-3

**Published:** 2017-12-28

**Authors:** Pavlina Capkova, Alena Santava, Ivana Markova, Andrea Stefekova, Josef Srovnal, Katerina Staffova, Veronika Durdová

**Affiliations:** 1 Department of Medical Genetics, University Hospital Olomouc, I.P.Pavlova 6, Olomouc, Czech Republic; 2 Department of Obstetrics and Gynaecology, University Hospital Olomouc, Olomouc, Czech Republic; 3 Institute of Molecular and Translational Medicine, Faculty of Medicine and Dentistry, Palacky University Olomouc, Olomouc, Czech Republic

**Keywords:** Microdeletion 14q22q23, Micrognathia, First trimester ultrasound, Pierre Robin sequence

## Abstract

**Background:**

Interstitial microdeletion 14q22q23 is a rare chromosomal syndrome associated with variable defects: microphthalmia/anophthalmia, pituitary anomalies, polydactyly/syndactyly of hands and feet, micrognathia/retrognathia. The reports of the microdeletion 14q22q23 detected in the prenatal stages are limited and the range of clinical features reveals a quite high variability.

**Case presentation:**

We report a detection of the microdeletion 14q22.1q23.1 spanning 7,7 Mb and involving the genes *BMP4* and *OTX2* in the foetus by multiplex ligation-dependent probe amplification (MLPA) and verified by microarray subsequently. The pregnancy was referred to the genetic counselling for abnormal facial profile observed in the first trimester ultrasound scan and micrognathia (suspicion of Pierre Robin sequence), hypoplasia nasal bone and polydactyly in the second trimester ultrasound scan. The pregnancy was terminated on request of the parents.

**Conclusion:**

An abnormal facial profile detected on prenatal scan can provide a clue to the presence of rare chromosomal abnormalities in the first trimester of pregnancy despite the normal result of the first trimester screening test. The patients should be provided with genetic counselling. Usage of quick and sensitive methods (MLPA, microarray) is preferable for discovering a causal aberration because some of the CNVs cannot be detected with conventional karyotyping in these cases. **To the best of our knowledge, this is the earliest detection of this microdeletion (occurred de novo), the first case detected by MLPA and confirmed by microarray**. Literature review of the genotype-phenotype correlation in similar reports leads us to the conclusion that dosage imbalance of the chromosomal segment 14q22q23 (especially haploinsuffiency of the genes *BMP4* and *OTX2*) contributes significantly to orofacial abnormalities. Association of the region with the Pierre Robin sequence appears to be plausible.

## Background

We report the detection of interstitial microdeletion 14q22q23 in a foetus with an abnormal facial profile in the first trimester of gestation and micrognathia, hypoplasia of the nasal bone and polydactyly in the second trimester of gestation discovered by ultrasound. Interstitial microdeletion 14q22q23 is a rare chromosomal syndrome associated with variable defects: microphthalmia/anophthalmia, pituitary anomalies, polydactyly/syndactyly of hands and feet, micrognathia/retrognathia. A growth restriction and developmental delay/mental retardation are common. Haploinsufficiency of the genes *BMP4* (*bone morphogenetic protein 4*) and *OTX2* (*orthodenticle homeobox 2*) is crucial for the majority of the phenotype features in the 14q22q23 microdeletion syndrome [1–5]. A prenatal ultrasound scan of these aberrations is less obvious and can be more difficult to distinguish or detect. In the case report, we describe the prenatal diagnosis of the interstitial microdeletion 14q22q23. **The first marker (abnormal facial profile) was observed during the ultrasound scan in the first trimester of pregnancy and has not been described previously.** We provide readers with a short review of the literature summarising clinical features of the aberration observed postnatally and prenatally. We focused on the orofacial malformations as a detectable feature for this abnormality in the early stages of pregnancy.

## Case presentation

**Fig. 1:**
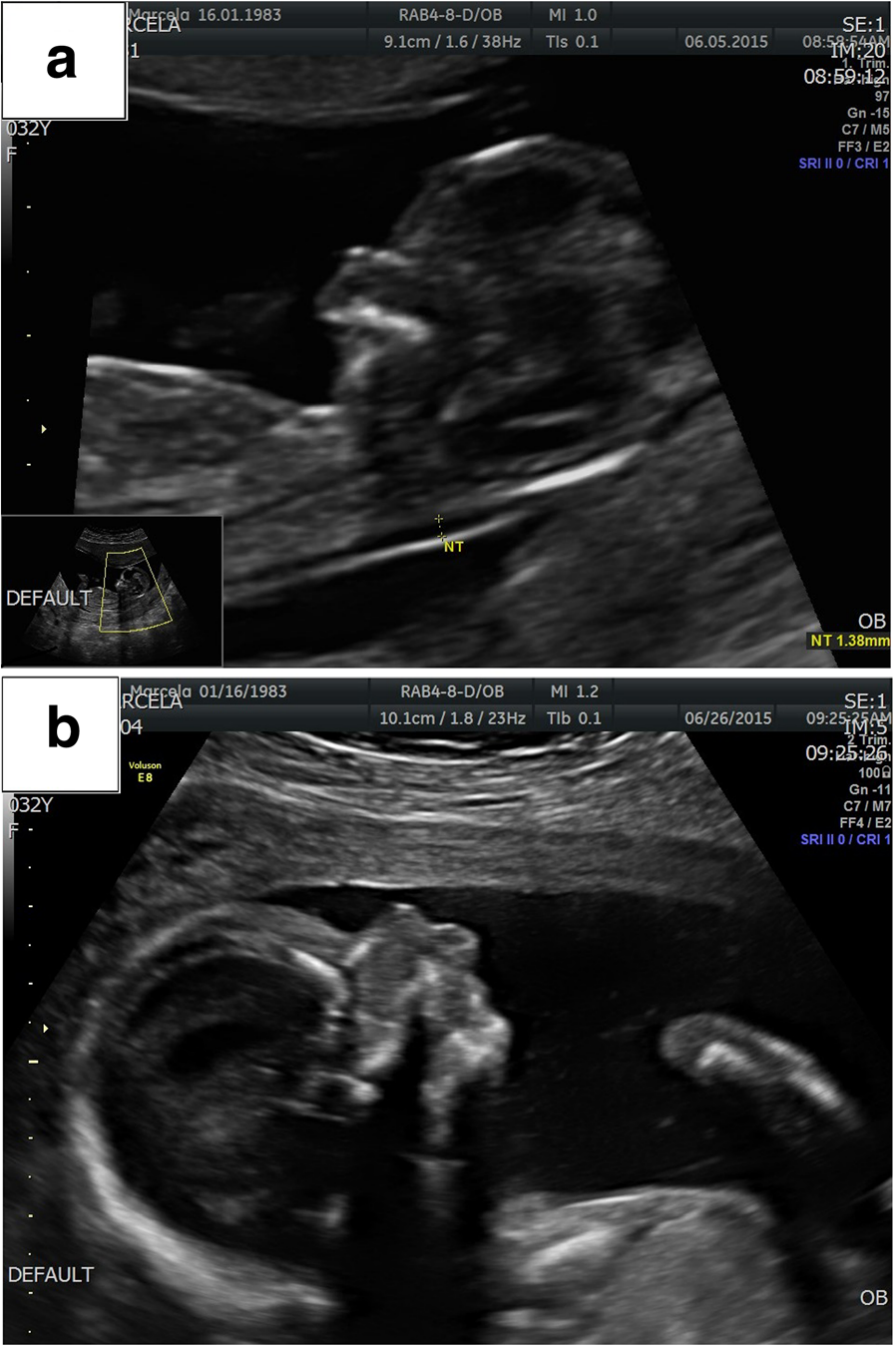
**a** Atypical foetal face profile in the first trimester of pregnancy. **b** Micrognathia and hypoplasia of the nasal bone in the second trimester of pregnancy

A 32-year-old pregnant woman (gravida 2, para 1) was referred to our department at 17th week of gestation for genetic counselling before amniocentesis recommended on the basis of ultrasound findings. The pregnancy was uneventful, the first trimester screening test, counting on the individual risk for trisomies 21, 13, 18, was with normal results. An abnormal facial profile of the foetus was observed (mandible hypoplasia – Pierre Robin anomaly or cleft lip were suspected) on the first trimester scan. Micrognathia was subsequently confirmed during the second trimester scan (17th week of the pregnancy). Bilateral postaxial polydactyly of the hands and hypoplasia of the nasal bone were also detected (Fig. 1). Both parents were non-consanguineous, the paternal age was 30, the first child – a girl – was healthy. The family history was unremarkable except for one case of a foetal congenital heart disorder in the child of a paternal aunt.

DNA was obtained from uncultured amniotic cells. A rapid molecular DNA analysis – QFPCR - excluded trisomies of the chromosomes 21, 18, 13. Conventional G-banding karyotyping was performed on metaphase mitotic cells obtained from amniotic fluid according to standard protocols. The karyotype of the foetus was 46,XY (500 bphs). MLPA testing with probemixes SALSA MLPA P036-E3 Subtelomeres Mix1 and P070- B3 Subtelomeres Mix 2B and P245 –B1 Microdeletion Syndromes 1 (MRC, Holland) were required for quick targeted exclusion of “common” microdeletions/microduplications. The DiGeorge syndrome was also excluded with probemix P311 – A2 CHD and P250-B2 DiGeorge syndrome (MRC, Holland) because of the micrognathia and family history of congenital heart defect. MLPA tests were performed according to the manufacturer’s protocols. Analysis of peak areas was performed using software Coffalyser (MRC, Holland).

The loss of the gene *BMP4* was revealed by probemix P311 in the foetus. This finding was confirmed by probemix P424-B2 CHD associated loci (MRC, Holland). Investigation of the parental samples of DNA did not reveal any changes. The loss was considered to be pathogenic. The parents opted for termination of pregnancy at 21st week of pregnancy. The extent of the deletion was specified by microarray (Affymetrix Cytoscan HD, Santa Clara, USA) using DNA extracted from the sample of foetal skin biopsy after TOP. Array data were analysed using Chromosome analysis suite (CHAS) software (Affymetrix, Santa Clara, USA).

The result was as follows: arr[GRCh37] 14q22.1q23.1[52468517_60202293]×1. The microdeletion spanned 7.7 Mb and involved 34 OMIM genes – 9 morbid genes (Table 1). The detected chromosomal aberration was concluded as a cause of the clinical findings in the foetus and occurred de novo. Performed autopsy revealed besides of micrognathia and polydactyly, bilateral anophthalmia and horizontal palpebral fissure, lack of gyrification of the brain. Other organs were found to be without any morphological abnormality. Parental karyotypes were: 46,XX and 46,XY.

**Table 1:** OMIM morbid genes included in the deleted region

Gene	Gene symbol	OMIM number
prostaglandin D2 receptor	PTGDR	604687
prostaglandin E receptor 2	PTGER2	176804
DDHD domain containing 1	DDHD1	614603
bone morphogenetic protein 4	BMP4	112262
GTP cyclohydrolase 1	GCH1	600225
transmembrane protein 260	TMEM260	617449
orthodenticle homeobox 2	OTX2	600037
KIAA0586	KIAA0586	610178
dishevelled binding antagonist of beta catenin 1	DACT1	607861

## Discussion

We report the microdeletion 14q22q23 detected in the prenatal case as such an early detection has not been reported previously. As cases of prenatal detection of the microdeletion are scarce, the comparison of the prenatally observed phenotype is limited. The relatively common features observed, however, in the few reported cases are orofacial malformations (micro−/rethrognathia, Pierre Robin sequence) which might be detected by US even in the first trimester of pregnancy as in our case [1, 2].

**Fig. 2:**
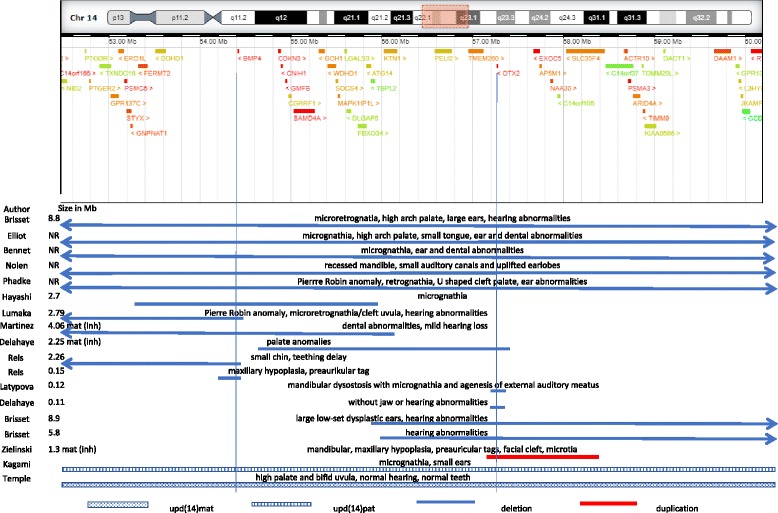
Overview of the relevant CNVs reported in the literature that overlap the interval of deletion detected in our patient. Abnormalities of structures evolved from the first pharyngeal arch are highlighted

In contrast to the scarce detection of the microdeletion in the prenatal stages, the discovery of the interstitial microdeletion postnatally has been more frequently reported. There are several genes in the interval of deletion that are intolerant to the loss of function or are likely to exhibit haploinsufficency (*DACT1, DDHD1, GCH1, BMP4, OTX2*) based on the data in Decipher. The loss of heterozygosity in two of them (*BMP4* and *OTX2*) is mostly consistent with the phenotype described in the patients. The hallmarks were ocular abnormalities [1–11] (Table 2). Absent or small orbits in the second trimester of gestation were seen [3, 7]. Bilateral anophthalmia was discovered during autopsy in our case. Considerable variability in particular ocular defects and limitations of US make detection of the microdeletion in foetus difficult particularly in the early stages of pregnancy. Intragenic mutations in *BMP4* and *OTX2* have been previously reported as having been causal for defects in ocular development [5, 8]. *BMP4* is expressed in optic vesicle and optic cup, brain, teeth and digits [5]. Brain anomalies: dilatation of the lateral ventricles [2, 10], volume loss of white matter in the brain [4, 5], agenesis of the corpus callosum or thin corpus callosum [4, 5, 7], hypoplasia or aplasia vermis [5, 8] were reported. Bilateral postaxial polydactyly of the hands [2, 9], syndactyly/polydactyly of the toes [4, 9, 10] were associated with the finding microdeletion 14q22q23 apart from abnormalities of the digits - clinodactyly, brachydactyly [1, 3, 4]. Postaxial polydactyly and a lack of brain gyrification were confirmed in this case. Pituitary anomalies or hypogonadism/hypothyroidism were observed in several reports [3–6, 11]. *BMP*4 expression is restricted to the diencephalic floor, which is consistent with the role in pituitary development [5]. Anomalies of the head were reported in the postnatal and prenatal cases [3–5, 7, 11]. Additional features associated with microdeletion involve micro−/retrognathia, maxillary hypoplasia, cleft uvula/palate, high arch palate or Pierre Robin sequence (PRS) [1–4, 8, 10–12] (Table 2). Micro−/retrognathia was seen during the US scan in the 17th week of pregnancy and orofacial abnormality was even noticed in the first trimester ultrasound scan in our case when the PRS in the fetus was suspected. The Pierre Robin (PRS) sequence can be isolated or observed as a feature of a syndrome. An association with gene loci 2q24.1-33.3, 4q32-qter, 11q21-23.1, and 17q21-24.3 has been found [13]. Micrognathia, glosoptosis, and cleft palate characterize PRS. Micrognathia and cleft palate are detected in individuals with *BMP4* haploinsufficiency [2, 10–12] (Table 2). The role of *BMP4* in otocephaly-agnathia has been previously implicated [14]. *BMP4* is expressed in the maxillary and mandibular processes [15]. Balanced BMP signalling and its local fine-regulation is critical for organizing and maintaining craniofacial tissues and can be one of the cause of the PRS [16, 17]. Referring to the role of BMP signalling in aetiology of PRS, one might also assume that the heterozygous loss of *BMP4* might be an underlying cause of the PRS. The dosage changes, however, in expression of *OTX2* also reveal the similar phenotype - mandibular, maxillary hypoplasia and retrognathia without ocular manifestation [18, 19] (Fig. 2). *OTX2* encodes a transcription factor that plays a critical role in craniofacial development and anterior brain morphogenesis. Heterozygous loss-of-function studies in mice showed a range of severe craniofacial anomalies – micrognathia, agnathia, anophthalmia - in haploinsufficient mice [20]. Micrognathia and ear anomalies were also observed in *SIX1* null murine mutants which indicate that *SIX1* might be another candidate for orofacial defects in the deleted interval [21]. However, *SIX1* was not involved in our detected deletion. It would seem that the contribution the genes of the region to the development of the mandible and ocular malformations might be more complex. It would be interesting to explore mutations in *BMP4*, *OTX2* or *SIX6 i*n patients with the isolated Pierre Robin sequence. The investigation has been focused on individuals with ocular manifestations thus far. *BMP4* is also required for normal endocardial cushion expansion and remodelling. Loss of *BMP4* in mice results in an insufficient number of cells in the developing outflow tract, endocardial cushions, defective cushion remodelling, ventricular septal defects, persistent truncus arteriosus, and abnormal semilunar valve formation [22]. Heart condition was reported only postnatally [9]. Some cases involving transposition of the great arteries, patent ductus arteriosus, and an atrioventricular canal defect can be found in the Decipher database. However, no heart condition was noted during the autopsy in our case. Homozygous mutations in gene *TMEM260,* positioned approximately 2.5 Mb from *BMP4,* are associated with renal, cardiac malformations and agenesis of corpus callosum [23], the defects are also present in microdeletion 14q22q23 or mutations in *BMP4* [4, 5, 7–9]. Interestingly, kidney malformation and jaw and ear defects are hallmarks of Townes-Brock syndrome 1- TBS1 (#107480) [24]. Recently, heterozygous mutation of *DACT1* has been reported as a cause of TBS2 (#617466) [25]. Hypospadia and ear abnormalities (microtia, overfolding of both ears), observed in patients with *DACT1* mutation, have been involved in the phenotype of the patients with microdeletion/duplication 14q22q23 [1, 3, 4, 11, 19]. Abnormality of jaws has not been involved, however, in the phenotype.

**Table 2:** Prenatal and postnatal clinical features in the particular cases of the microdeletion 14q22q23

Author	This Report	Bennet [1991]	Nolen [2006]	Phadke [1994]	Pearce [2012]	Brisset [2014] (Pat 1)	Thienpont [2007]	Bakrania [2008]		Lumaka [2012]				Hayashi [2008]	Reis [2011]		Martínez-Fernández [2013]		Delahaye [2012]		Latypo-va [2016]	Brisset [2014]	
genes relevant to the phenotype	BMP4 and OTX2									BMP4										OTX2			
age	TP	TP	neonate	neonate	neonate	4Y (Pat. 1)	NR	NR (Pat. 1)	NR (Pat. 2)	familial				1Y	6Y (Pat. 1)	12Y (Pat. 2)	familial		24 Y (Pat. 3)	14Y (Pat. 4)	27Y	4Y (Pat. 2)	4Y (Pat. 3)
size in MB	7.7	NR	9.66	NR	5.7	8.8	6.7	NR		2.79				2.7	2.2	0.15	4.06		2.25	0.11	0.12	8.9	5.8
gender	M	F	M	M	F	F	M	M	F	F	F	F	M	F	F	F	F	F	M	F	F	F	M
microphtalmia	–	–	–	–	+	–	–	–	–	+	–	–	–	–	Rieger anom.	+	–	–	+	+	–	–	–
anophtalmia	+/autopsy	+	+	+	+	+	+	+	+	–	–	–	–	–	–	–	–	–	–	–	–	+	+
sclerocornea	–	NR	–	–	–	–	–	–	–	+	–	–	–	+	–	–	–	–	–	–	–	–	–
poly−/syndactyly of hands	+	–	+	–	–	+	+	–	–	+	–	–	–	–	–	–	–	+	–	–	–	–	–
poly−/syndactyly foot	–	–	+	–	–	–	–	–	–	–	+	–	+	+	–	–	–		–	–	–	–	–
digit abnormality (−dactyly)	–	brachy	brachy	clin	–	–	–	NR	NR	NR	NR	NR	NR	–	–	–	–	–	–	–	–	–	–
micro/rethrogna-thia	+	+	+	+	–	+	–	–	–	+	–	+	–	+	small chin	maxillary hypoplas.	–	–	–	–	+	–	–
cleft uvula/palate	–	–	–	+	oral frenulum	–	–	–	–	+	–	+	–	–	–	–	–	–	+	–	–	–	–
hearing/ear anomalies	NR	+	+	+	+	+	+	+	–	–	–	+	–	–	–	+	–	–	–	–	+	+	+
brain anomalies	+/autopsy	NR	+	–	–	+	–	+	+	+	–	+	–	+	–	–	–	+	–	–	–	–	–
ID or DD	NR	NR	+	+	NR	+	–	+	+	+	–	+	–	+	+	+	NR	+	+	+	–	+	+
CHD	–	–	–	–	–	+	+	–	–	–	–	–	–	–	–	–	–	–	–	–	–	–	–
anomaly of head (−cephaly)	–	micro-	brachy−/micro-	micro-	micro-	–	–	plagio−/micro-	–	–	–	NR	NR	–	makro	–	makro	–	micro	–	–	micro	–
cryptorchidism/ hypospadia	NR	NR	+	hypo-spadia	NR	–	+	+	–	–	–	–	–	–	–	–	–	–	–	–	–	–	testicu-lar reten.
growth delay	–	NR	+	low birth weight	–	+	NR	NR	NR	+	+	+	–	+	+	–	–	–	–	+	–	+	+
pituitary hypoplasia	NR	+	+	–	–	+	NR	+	hypothyr.	NR	NR	–	NR	–	NR	NR	–	–	–	+	–	+	+
week of detection	12w/17w	21w	32 w	–	2nd trimester	–	–	–	–	18w	–	–	–	–	–	–	–	–	–	–	–	–	–
antenatal findings	microgna-thia poly-dactyly of hands, hypop.of the NB, susp. Pierre-Robin anomaly	increased NT, unusual shape of head, small orbits	IUGR	Pierre-Robin anomaly	polyhydr-amnion absent orbit, duodenal atresia, deficient corpus callosum					retrognatia, absent NB, susp. Polydactyly, Pierre-Robin anomaly				LB - liveborn, TP – termination of pregnancy, NB - nasal bone, US - ultrasound NR - not reported, (+) presence of the clinical feature, (−) absence of the feature								IUGR	
outcome of pregnancy	TP	TP	LB	LB	LB					LB

Taken all together, deletions covering the interval between the genes *BMP4* and *SIX6* are associated with a typical phenotype with ocular, orofacial and brain malformations, growth delay, pituitary anomalies and poly−/syndactyly. Smaller deletions, involving either of the genes, are manifested with milder phenotypes. Deletions, involving *BMP4* and adjacent genes without *OTX2,* have been suggested recently as the cause of Frias syndrome – growth deficiency, exophtalmia, facial anomalies (cleft lip and palate, palpebral ptosis) and hand and foot alternations [26]. Moreover, deletions covering *OTX2* are more frequently associated with microcephaly [1, 7, 11, 12] in comparison with the mutations restricted to *BMP4* where macrocephaly has predominantly been reported [8, 26]. Extreme variability in phenotypes, either large or smaller deletions, indicates incomplete penetrance and the modifying effect of genetic background (interplay of other genes in different or common molecular pathways such as *SHH, FGF8*, etc) [14, 27]. Little is known about the influence of the parental origin of the deletion on the resulting phenotype. It should be taken into account, however, as the upd (14) either paternal or maternal, causes recognizable syndromes. There are certain features which overlap with the phenotype of the microdeletion 14q22q23. Growth restriction, for example, in patients with upd (14) mat [11, 28], polyhydramnion in patients with upd (14) pat [7, 29] or orofacial malformations in both. Micrognathia, retrognathia or high arch palate are frequently described in patients either with upd (14) mat [28] or upd (14) pat [30–32]. Paternal isodisomy of chromosome 14 was described in patients with maxillary and mandibular hypoplasia [33]. Eggermann has reported the case of segmental upd (14) mat ranges from 14q12 to 14q31. The propositus revealed hypoplastic and retracted mandible, but no ocular, brain or other congenital malformations [34]. Moreover, there is some evidence that maternally inherited duplication of *OTX2* manifested with hemifacial microsomia involving mandibular, maxillary hypoplasia and retrognathia [19]. It might indicate that equal biparental contribution of genes *BMP4* or *OTX2* is necessary for correct maxillary and mandibular development. Recently, the cluster of genes in interval 14q32.2, including paternally expressed genes *DLK1* and *RTL1* and maternally expressed genes *MEG3* (*GTL2*), *RTL1* (RTL1 antisense), *MEG8*, snoRNAs, and microRNAs, has been identified as responsible for imprinting disorders: Temple syndrome (#616222) and Kagami - Ogata syndrome (**#** 608149) [35]. It is not clear whether expression of further genes on chromosome 14 is influenced by imprinting.

## Conclusion

We assume that the heterozygous loss of genes *BMP4* and *OTX2* determines the major features detected in the foetus. Both genes are relevant in the development of craniofacial structures (especially structures evolved from the first pharyngeal arch). The phenotype of the microdeletion is highly variable even within families, which implies variable penetrance. If an atypical foetal face profile is found during the ultrasound scan in the first trimester of pregnancy, it can be a sign of rare chromosomal abnormalities. If the micro−/retrognathia or Pierre Robin sequence is observed during ultrasound scan in the second trimester of pregnancy, the presence of micro- or anophthalmia should be checked as a possible marker of microdeletion 14q22q23. The patient should consult the diagnosis with the clinical geneticist. Molecular karyotyping should be used in the cases. However, method MLPA might also provide the useful and cost-efficient alternative for detection of such an abnormality, where mandibular, ophthalmic or orofacial abnormalities are prenatally seen.

## Abbreviations

CNV: Copy number variant
IUGR: Intrauterine growth retardation
MLPA: Multiplex ligation-dependent probe amplification
TOP: Termination of pregnancy
